# Metastatic Calcinosis Cutis: Unusual Locations in End-Stage Renal Disease

**DOI:** 10.7759/cureus.53835

**Published:** 2024-02-08

**Authors:** JC Charm C Asoy, Justin Luke D Yap

**Affiliations:** 1 Department of Radiology, Northern Mindanao Medical Center, Cagayan de Oro, PHL

**Keywords:** ultrasonography, hemodialysis, computed tomography, end-stage renal disease, metastatic calcinosis cutis

## Abstract

Metastatic calcinosis cutis is a rare consequence of end-stage renal disease (ESRD), which occurs due to elevated levels of serum phosphorus and abnormal phosphate and calcium metabolism, leading to the precipitation and deposition of calcium in the cutaneous and subcutaneous tissues. This paper reports the case of a 33-year-old male with ESRD and a six-year history of hemodialysis treatment who presented with multiple areas of gradually enlarging, lobulated calcified soft tissue masses observed bilaterally at the level of the acromioclavicular joint and superomedial aspect of the right thigh, extensively involving the perineal region and the right superior anterior chest wall. The unique character of this case is the rare involvement of the sternoclavicular joint and the symphysis pubis. The relevant laboratory findings included elevated levels of serum phosphorus, blood urea nitrogen, and creatinine, which were consistent with metastatic calcinosis cutis as a consequence of ESRD. The treatment of secondary calcinosis cutis primarily includes low-calcium and low-phosphorus diets, dialysates, and phosphate binders, except aluminum-containing binders, which were advised for this patient. Imaging is the mainstay for the diagnosis of calcinosis cutis, and as metastatic calcinosis cutis is an infrequent and debilitating consequence of ESRD, prompt diagnosis and appropriate treatment are paramount.

## Introduction

Calcinosis cutis is a state where calcium phosphates are deposited in the skin and subcutaneous tissues. Metastatic calcification is a subcategory of calcinosis cutis, which results from abnormal calcium and/or phosphate metabolism, as an uncommon complication of end-stage renal disease (ESRD) [1-3]. Other subcategories include dystrophic calcification, idiopathic calcification, iatrogenic calcification, and calciphylaxis [2].

Metastatic calcinosis cutis is a unique radiographic finding but not totally rare in patients with ESRD. It presents with multiple calcified periarticular tumors commonly involving the extensor surfaces of the hip, elbow, shoulder, foot, and wrist joints [2,3]. There are very few reports documenting the involvement of the sternoclavicular joint and symphysis pubis in patients with calcinosis cutis. We present a case of metastatic calcinosis cutis extensively involving the shoulder joints, superomedial aspect of the right thigh, sternoclavicular joint, and symphysis pubis.

This article was previously presented as a meeting abstract at the 79th Korean Congress of Radiology on September 20-23, 2023.

## Case presentation

We present the case of a 33-year-old man with ESRD and a six-year history of hemodialysis treatment. The patient has no history of previous trauma, cancer, or granulomatous illness but presented with multiple areas of lobulated, non-tender, calcified soft tissue masses observed bilaterally at the level of the acromioclavicular joint and superomedial aspect of the right thigh, extensively involving the perineal region and the right superior anterior chest wall. These masses were first observed three years into the patient's hemodialysis treatment and were noted to be gradually enlarging. 

Pertinent laboratory findings included elevated levels of serum phosphorus (8.82 mg/dL; normal range: 2.5-4.5 mg/dL), blood urea nitrogen (49 mg/dL; normal range: 8.4-25.7 mg/dL), and creatinine (6.04 mg/dL; normal range: 0.6-1.8 mg/dL). The findings were in alignment with metastatic calcinosis cutis as a consequence of ESRD. The advised treatment for this patient included low-calcium and low-phosphorus diets, dialysates, and phosphate binders, except aluminum-containing binders, since studies have shown that long-term use has led to bone disease, encephalopathy, and anemia. The patient is expected to follow up within the next six months, and monitoring of the calcified soft tissue masses will be performed.

**Figure 1:**
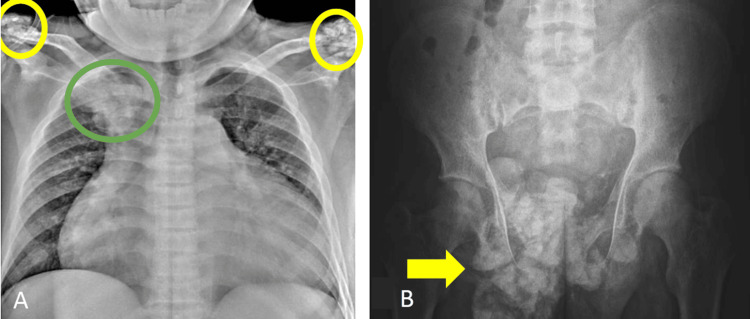
Radiograph of the chest and pelvis Chest radiograph (A) showing lobulated density projecting over the right upper hemithorax (green circle) measuring 5.2x6.4 (CCxW). Similarly, multifocal calcifications are observed in the bilateral acromioclavicular joint regions (yellow circles) measuring 4.5x4.8 cm (CCxW) in the right and 3.8x4.8 cm (CCxW) in the left. Pelvic radiograph (B) showing multilobulated calcifications seen in the right pelvic region (arrow) measuring 12.2x11.3 cm (CCxW).

A series of radiographs (Figure 1) showed amorphous, multilobulated calcifications within the soft tissues.

**Figure 2:**
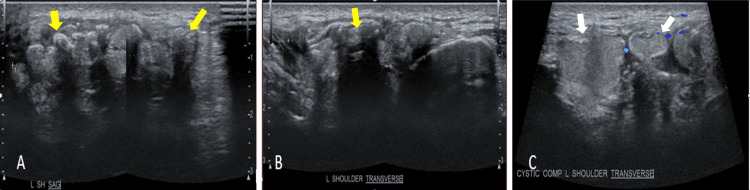
Ultrasound of the left shoulder (A, B) Well-defined complex masses predominantly composed of multiple echogenicity exhibiting posterior acoustic shadowing (yellow arrows) and areas of (C) cystic foci with midlevel echoes exhibiting Brownian motion upon compression (white arrows). The mass has a conglomerate measurement of about 5.1x4.3x1.5 cm (CCWAP).

**Figure 3:**
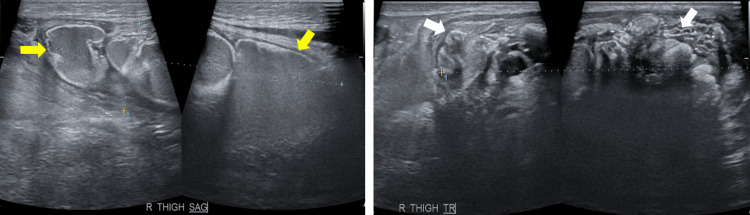
Ultrasound of the right superomedial thigh mass Ultrasound of the right superomedial thigh mass showing cystic (yellow arrows) and solid (white arrows) components of the mass. The mass has a conglomerate measurement of about 9.4x7.7x2.4 cm (CCWAP).

The sonographic evaluation (Figure 2 and Figure 3) revealed hyperechoic shadowing foci reflective of the calcified nature of the masses in the right clavicular region, bilateral shoulders, and right thigh.

**Figure 4:**
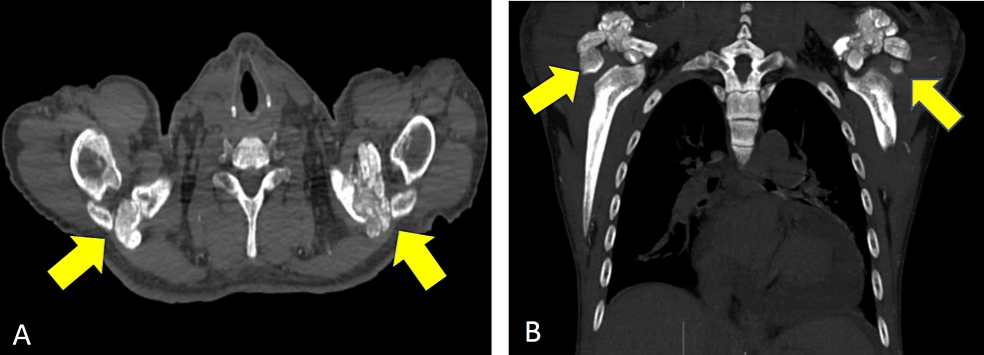
Plain CT of the chest (A) Axial and (B) coronal views showing amorphous, multilobulated masses in both acromioclavicular joints (arrows). The masses measure 4.5x4.1x2.8 cm (CCWAP) in the right and 3.5x4.8x4.3 cm (CCWAP) in the left. CT: computed tomography

**Figure 5:**
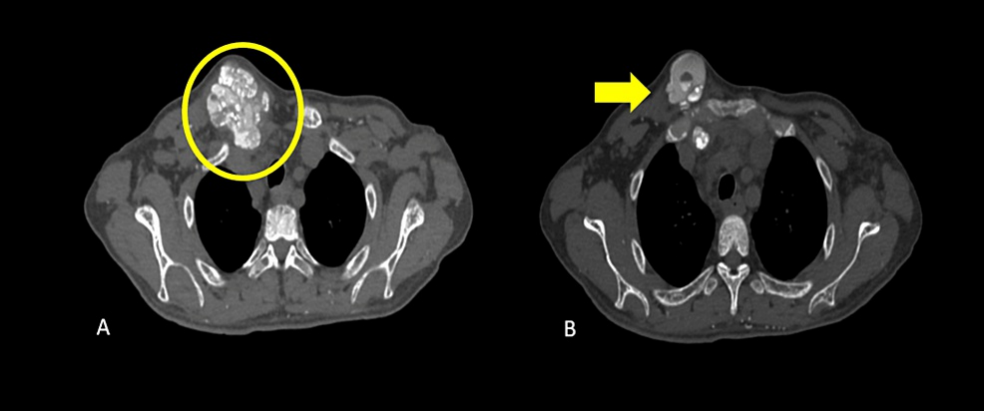
Plain CT of the chest Axial (A, B) views showing multilobulated, calcified lesions of the right sternoclavicular joint (circles), with a few demonstrating sedimentation (arrow). The mass measures 4.5x6.2x6 cm (CCWAP). CT: computed tomography

**Figure 6:**
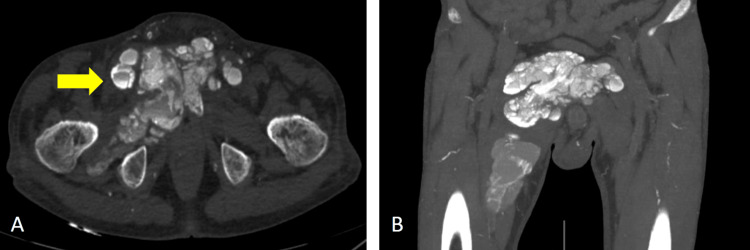
Axial and coronal plain CT scan of the pelvic region Multilobulated calcified mass with areas demonstrating sedimentation (arrow) measuring 12x11.6x9 cm (CCWAP). CT: computed tomography

**Figure 7:**
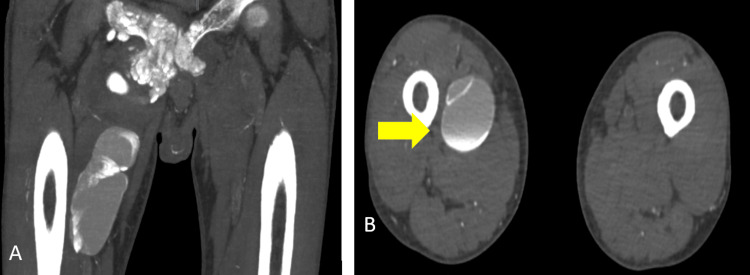
Plain CT scan of the lower extremities Large cystic mass with fluid-calcium layering or sedimentation sign in the right upper inner thigh measuring 11.6x4.3x4.6 cm (CCWAP). CT: computed tomography

A nonenhanced computed tomography (CT) of the chest and whole abdomen (Figure 4, Figure 5, Figure 6, and Figure 7) were obtained showing lobulated inhomogeneous calcified lesions with cystic components, some of which demonstrated fluid-calcium levels or sedimentation confined to the soft tissue spaces with a note of osseous involvement of the right superior pubic ramus.

## Discussion

Metastatic calcinosis cutis occurs due to abnormal calcium and/or phosphate metabolism that leads to the precipitation of calcium in the cutaneous and/or subcutaneous tissues [1,2]. It is characterized by lobular, densely calcified masses confined to the soft tissues, commonly at the extensor surface of the joint in the anatomic distribution of the bursa [1-4]. The prevalence of calcinosis cutis in patients with ESRD is approximately 1% (0.5-1.2%). It occurs after a short period of dialysis with a median period of four (1-7) years [1,5]. Massive development of such calcifications may limit physical function and negatively impact the patient's quality of life [6].

Alberto Inclan, though not the first to characterize the disease, coined the term "tumoral calcinosis" in 1943 [1]. Several terms have been used to describe this condition, including "uremic tumoral calcinosis," "secondary tumoral calcinosis," "pseudotumor calcinosis," and "tumoral calcinosis-like lesion" [1].

The disease has been associated with high levels of serum phosphorus, as a consequence of chronic renal failure. Precipitation can occur when levels of calcium and phosphate reach a solubility threshold [6]. However, the exact mechanism for the occurrence of these massive periarticular calcifications remains unknown [1]. Although elevated serum phosphorus levels may be necessary, they are solely insufficient to explain the unusual prevalence of massive periarticular calcifications in individuals with uremia [1,5].

The common radiographic findings include lobulated calcified mass within the soft tissues, which is typically cystic, has bursal distribution, and usually affects the extensor surfaces. The common sites of distribution in descending order are as follows: hip, elbow, shoulder, foot, and wrist. Other cases have also shown involvement of the temporomandibular joint, scalp, larynx, spine, sacrum, hand, and knee [1,5].

The diagnosis involves a combination of radiographic evidence of the disease alongside laboratory data and pertinent medical history [4]. CT is the imaging modality of choice to delineate the calcified lesions for the purpose of surgical planning [5]. The characteristic fluid-calcium levels observed within the cystic masses caused by calcium layering are known as the sedimentation sign. Another finding that characterizes this disease is the absence of erosion or osseous destruction by the adjacent soft tissue mass. One differential diagnosis which presents similarly is tumoral calcinosis, a hereditary familial condition [1]. However, few cases have shown destruction and involvement of the adjacent bone, possibly caused by recurrent microtrauma due to mass effect and periarticular location. For cases involving bone destruction, one differential consideration would be chondrosarcoma. The sedimentation sign, a pathognomonic finding, will lead to the diagnosis of calcinosis cutis, if present [7]. The detection of loculated fluid collection on ultrasound helps in determining the disease activity. The cystic appearance of lesions is suggestive of increased metabolic activity. Compared with cystic lesions, solid homogeneous lesions tend to possess lesser metabolic activity and development potential [1,5].

The treatment of secondary calcinosis cutis primarily includes low-calcium and low-phosphorus diets, dialysates, and phosphate binders, except aluminum-containing binders. Surgical excisions and interventions tend to have more complications, such as infection and fistula formation [3]. However, subtotal parathyroidectomy or renal transplant is a possible treatment option for those with poor response to medical management [4].

## Conclusions

Metastatic calcinosis cutis are calcified masses, which involve the extensor surfaces of the joints and commonly affect the hip, elbow, shoulder, foot, and wrist joints. The unusual involvement of the sternoclavicular joint and the symphysis pubis is the basis of this case. As metastatic calcinosis cutis is an infrequent and debilitating consequence of ESRD, prompt diagnosis and appropriate treatment are crucial. Several disease entities can possess similar appearances upon imaging; thus, it is imperative to have a multimodality radiologic approach. The familiarity and clinical correlation ability of the radiologist play a critical role in the treatment and management of such uncommon pathologies.
